# The role of resilience in the relationship between adverse childhood experiences and psychosis and bipolar symptoms

**DOI:** 10.1192/bji.2025.10087

**Published:** 2026-11

**Authors:** David M. Ndetei, Victoria Mutiso, Christine Musyimi, Samuel Walusaka, Veronica Onyango, Eric Jeremiah, Pascalyne Nyamai, Kamaldeep Bhui, Daniel Mamah

**Affiliations:** 1 Africa Institute of Mental and Brain Health, Nairobi, Kenya; 2 Department of Psychiatry, University of Nairobi, Nairobi, Kenya; 3 World Psychiatric Association Collaborating Centre for Research and Training, Nairobi, Kenya; 4 Department of Psychiatry, Nuffield Department of Primary Care Health Sciences, University of Oxford, Oxford, UK; 5 World Psychiatric Association Collaborating Centre, Oxford, UK; 6 Washington University School of Medicine, St Louis, MO, USA

**Keywords:** Adverse childhood experiences (ACEs), psychosis, bipolar symptoms, resilience, Kenyan youth

## Abstract

**Background:**

Adverse childhood experiences (ACEs), including abuse, neglect and household challenges, have been linked to various mental health outcomes, including psychosis and bipolar symptoms. Research indicates a 57–80% increased risk of psychosis among individuals exposed to ACEs.

**Aim:**

This study examines the relationship between ACEs and psychotic/bipolar symptoms in Kenyan adolescents, assessing resilience as a potential moderator or mediator.

**Method:**

This cross-sectional study was conducted in Nairobi and Kiambu counties, Kenya. A total of 1972 youth, aged 14–25 years, were recruited and completed questionnaires focusing on ACEs (Trauma and Distress Scale), psychosis/bipolar symptoms (Washington Early Recognition Center Affectivity and Psychosis screen) and resilience (Adult Resilience Measure +16). Descriptive statistics, Pearson correlation and multiple linear regression were used to assess the relationships between ACEs and psychosis/bipolar symptoms. SPSS Process macro was employed to examine the moderating/mediating role of resilience.

**Results:**

Emotional abuse and physical abuse were significantly associated with higher psychosis and bipolar symptoms (*P* < 0.001). Emotional neglect negatively impacted bipolar symptoms (*P* = 0.042). Resilience moderated the relationship between sexual abuse and psychosis, but not bipolar symptoms. Mediation analysis showed that resilience partially mediated the relationships between sexual abuse and both psychosis and bipolar symptoms.

**Conclusions:**

ACEs, particularly emotional and physical abuse, are significant predictors of psychosis and bipolar symptoms in Kenyan youth. Resilience may play a key role in moderating and mediating these relationships, particularly in cases of sexual abuse. Developing resilience-focused interventions could help mitigate the long-term effects of ACEs on mental health.

Adverse childhood experiences (ACEs) encompass a range of adversities and traumatic events, including abuse (physical, emotional or sexual), neglect (emotional or physical) and household challenges (substance abuse, domestic violence, parental separation/divorce, incarcerated parent or mental illness)^1,2^ (see Fig. 1). ACEs can lead to altered neurodevelopment and numerous pan-diagnostic symptoms including emotional dysregulation, impulsivity, anxiety and depressive feelings, as well as marked difficulties in forming and sustaining relationships.^3^ There is a change in the function of the hypothalamic–pituitary–adrenal axis, resulting in altered stress responses^4^ and long-term health consequences.^5^ ACEs can result in the development of many mental disorders including post-traumatic stress, anxiety, depression and psychosis.^6^

**Fig. 1:**
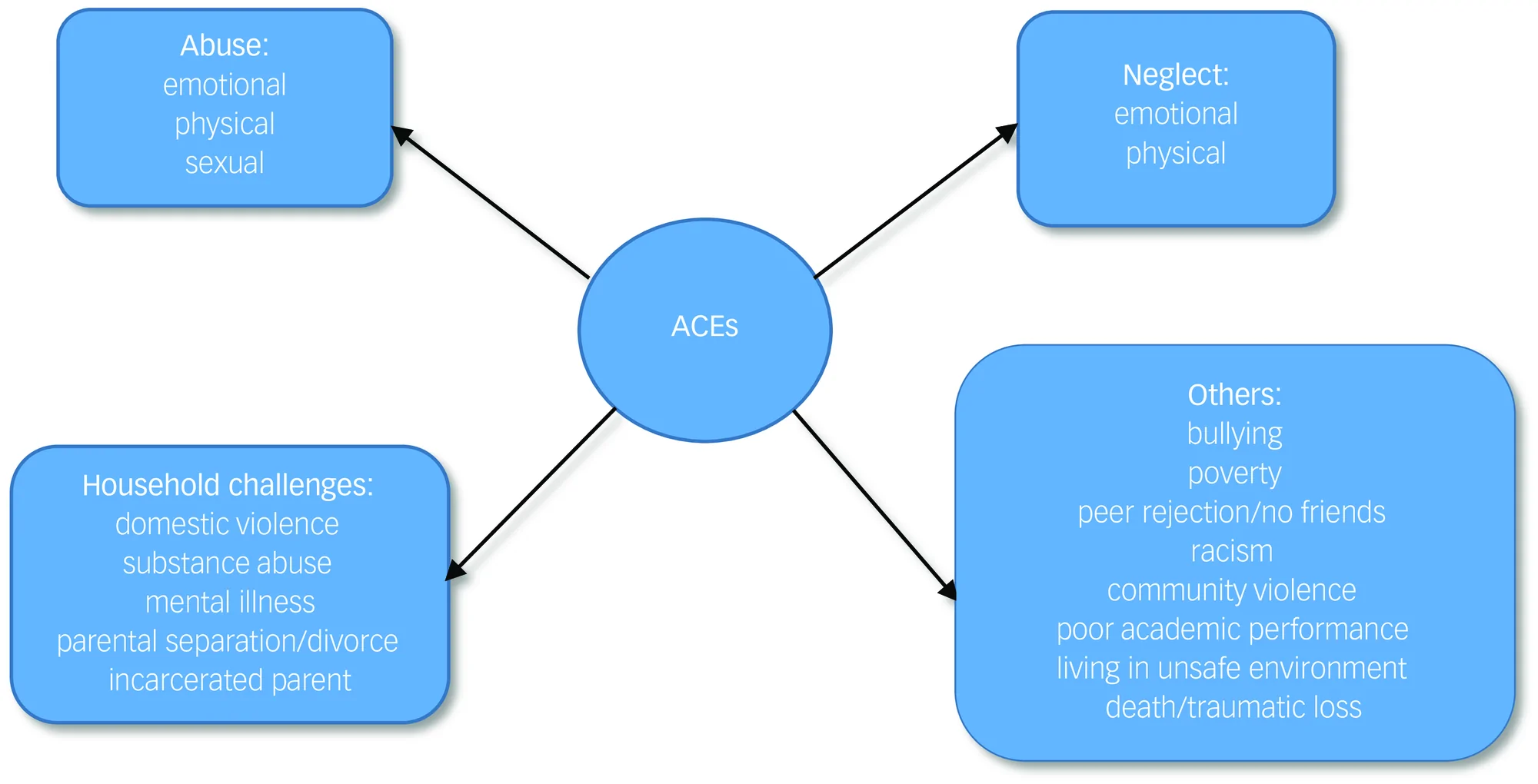
Different categories of adverse childhood experiences (ACEs).

## Psychosis and clinical spectrum

Psychosis, as defined by the American Psychiatric Association, entails disturbances in thoughts, emotions and perceptions, representing a severe mental health condition often associated with impaired social functioning.^7^ Psychosis diagnoses include psychotic spectrum disorders (delusional disorder, schizophrenia and schizoaffective disorder) and mood spectrum disorders (bipolar disorder).^8,9^

## Association between ACEs and psychosis

A range of meta-analyses and studies suggest that exposure to ACEs is associated with an increased risk of developing psychosis or related disorders later in life, such as schizophrenia and bipolar symptoms.^10,11^ In the UK, studies have shown a strong link between ACEs and the development of psychosis and bipolar disorder among adolescents, with emotional abuse (*r* = 0.33) and neglect being significant risk factors. Physical abuse was more strongly associated with schizotypy in women, while sexual abuse had a stronger impact on youth.^12,13^ In the African context, studies in Sudan, South Africa, Kenya and Uganda have demonstrated a strong association between ACEs and the development of psychosis and bipolar symptoms among adolescents.^11^ Understanding the mechanisms by which ACEs lead to psychosis, and also what might prevent the development of psychosis, could lead to the development of preventive interventions.

### The role of resilience

Resilience, defined as the ability to adapt and recover from adversity, has been shown to moderate the impact of ACEs on the development of psychotic symptoms. Research suggests that higher levels of resilience can buffer the negative effects of ACEs, reducing the likelihood of psychiatric symptoms such as psychosis and bipolar symptoms.^14,15^ While prevention of ACEs remains the ultimate public health goal, our study focuses on resilience as a secondary protective mechanism, mitigating the psychological consequences of ACEs when prevention has not occurred.

## Knowledge gap

There is a knowledge gap regarding the mechanisms by which ACEs lead to the development of psychosis, and also regarding the role of resilience as either a moderator or mediator. In order to improve prevention in Kenya, we undertook studies of ACEs and psychosis, and the role of resilience in Kenyan adolescents living in the Nairobi metropolitan area. Although the Nairobi metropolitan is largely urban, it hosts youth from diverse socioeconomic and ethnic backgrounds across Kenya, offering a microcosm of national experiences. However, generalisation to rural populations should be made cautiously.

## General aim

The general objective of this study is to examine the association between ACE scores and psychotic and bipolar symptoms among Kenyan youth in the Nairobi metropolitan area, which is a microcosm of the overall Kenyan population.

## Specific aims

- to assess the prevalence of ACEs among youth in the Nairobi metropolitan area;
- to examine the relationships between ACEs and both psychosis and bipolar disorder;
- to evaluate whether resilience has a moderating or mediating role in the relationships between ACEs and both psychosis and bipolar disorder.

## Method

### Study design and setting

This was a cross-sectional study covering two counties in the Nairobi metropolitan area, i.e. Nairobi and Kiambu counties. Nairobi is the capital city and an urban economic hub, while Kiambu is a mix of urban and rural areas. Students from a middle-level college and a medical training college located within the metropolitan area were also recruited into the study.

### Sampling and inclusion/exclusion criteria

The study’s inclusion criteria were youth aged 14–25 years and residing within the designated neighbourhoods. The exclusion criteria were individuals outside the selected age range; those incapable of comprehending the questionnaire due to factors such as intoxication or illiteracy; those unwilling to participate; those who did not consent to the study; and those under the age of 18 years, regarded as minors in Kenyan law, whose parents/guardians did not give consent for them or who did not assent to the study despite a full explanation of the study. However, there is a caveat to this in that those with psychosis had a reduced capacity to give informed consent.

### Procedures

#### Research assistant selection and training

Twelve research assistants were selected through a competitive hiring process to assess their previous experiences and interpersonal skills. Subsequently, successful candidates underwent a comprehensive, 2-day, in-person training session. This training not only covered data collection techniques and protocols relevant to the study, but also incorporated mock interviews to simulate real-world scenarios in community engagement, consent and assent explanation and the consenting and assenting process, including the right to withdraw at any time without covert victimisation.

#### Recruitment and resulting sample

All data were collected in the daytime from 21 September to 15 December 2022, based on a schedule that also accommodated a crucial sensitisation phase through existing local networks. We approached the County Commissioners in Kiambu and Nairobi counties and, following their approval, linked with chiefs and sub-chiefs who worked in collaboration with elders to sensitise and mobilise youth in their areas. This detailed community engagement ensured that those attending for data collection were already included in the study. A total of 1972 participants who participated provided written informed consent/assent, thereby affirming their voluntary participation according to the ethical guidelines.

#### Instruments

- Sociodemographic profile: sociodemographic data were obtained from participants using a self-reported questionnaire to gather information on age, gender, marital status, religion, birth position, level of education, employment status, primary source of income, place of abode and whether they were sharing living space(s).
- The Traumatic and Distress Scale (TADS): TADS is a self-report questionnaire designed to assess a broad range of childhood traumas and distressing experiences.^16^ It captures ACEs across five main domains: emotional abuse, physical abuse, sexual abuse, emotional neglect and physical neglect. Each item is rated on a 4-point Likert scale from 1 to 4 (0 = never, 1 = rarely, 2 = sometimes, 3 = often, 4 = nearly always). TADS has excellent internal consistency in every domain and has been proved to be a valid, reliable and clinically useful instrument for assessment of retrospectively reported childhood traumatisation in a general population sample.^16^ For this study, total TADS scores were computed across all five domains to quantify overall childhood trauma severity.
- Washington Early Recognition Center Affectivity and Psychosis (WERCAP) screen: the WERCAP screen is a self-report instrument developed to identify individuals at risk for psychosis or bipolar disorder.^17^ It assesses the presence and severity of subthreshold psychotic-like and affective (bipolar-like) symptoms, and also associated functional decline. Unlike diagnostic tools, WERCAP focuses on early symptom patterns and risk states rather than confirmed clinical diagnoses. The screen assesses functional decline, subthreshold psychotic symptoms and associated affectivity.^18^ WERCAP is a validated tool that can be rapidly deployed at the population level to identify high-risk individuals who require further assessment. We used the WERCAP screen to assess symptoms in the preceding 3 months. A previous validation study in a community setting in Kenya reported an excellent area under the receiver operating characteristic curve of 0.83 against the Structured Interview for Psychosis-Risk Syndromes, a gold standard in the diagnosis of high-risk syndromes for psychosis.^19,20^
- Adult Resilience Measure +16 (ARM-R): ARM-R is a self-report questionnaire designed to assess resilience through a socioecological lens.^21,22^ It evaluates an individual’s capacity to adapt to, and recover from, adversity through access to personal, relational and contextual resources. Comprising 17 items, respondents rate each item on a 5-point Likert scale ranging from ‘Not at all’ (1) to ‘A lot’ (5). ARM-R measures both personal resilience, which pertains to an individual’s ability to access resources in their environment, and relational resilience, which evaluates the support provided by social entities including family, peers and institutions. All items are positively framed, making scoring straightforward by summing the scores directly. Total scores on ARM-R range from 17 to 85, with higher scores indicating stronger resilience-related traits. Developed through a cross-cultural study involving 14 communities across 11 countries,^23^ ARM-R demonstrates good psychometric properties, including strong internal reliability/consistency, content and face validity, and is used across cultures in diverse studies of resilience.^22,23^

#### Data collection

Data were collected on TADS, resilience and WERCAP, together with accompanying sociodemographics. These questionnaires were self-administered by participants in a group setting in social halls within the study sites. The first aspect of this process entailed random assignment of a number (1–12) to a research assistant, with each number forming a distinct group. Participants were then assigned to their group in a restricted randomisation, where a printed voucher with numbers 1–12 on each page was used to ensure that group sizes were balanced. Each group comprised a maximum of 25 participants and was led by the respective research assistant assigned to that group. The research assistants facilitated the distribution of the questionnaires and collected written informed consent/assent upon these being signed. They also cross-checked the participants’ ages to ensure compliance with the inclusion criteria. To ensure standardisation, participants were instructed to respond to the questions based on their understanding, thereby minimising any potential influence from the research assistant.

### Ethical standards

The authors assert that all procedures contributing to this work comply with the ethical standards of the relevant national and institutional committees on human experimentation, and with the Helsinki Declaration of 1975 as revised in 2008. All procedures involving human subjects/patients were approved by the Nairobi Hospital Ethics Research Committee (approval no. TNH-ERC/DMSR/ERP/022/22). The study obtained licensing from the National Commission for Science, Technology and Innovation (NACOSTI; licence no. NACOSTI/P/22/18097). Administrative permissions were also sought from the county-level offices in Kiambu and Nairobi counties, as well as institutional approval from the colleges. Informed written consent/assent was obtained from participants before data collection commenced. For participants younger than 18 years, informed written consent to participate was obtained from their parents or legal guardians.

### Statistical analysis

All statistical procedures were reviewed by a biostatistician, to ensure accuracy and appropriate model selection. Analysis was performed using the Statistical Package for the Social Sciences (IBM SPSS Statistics version 25 for Windows). The frequency distributions and prevalences of different ACEs were computed. Descriptive statistics summarised the central tendency (mean), dispersion (standard deviation) and distribution (skewness) of ACEs and WERCAP scores. The analysis sought to determine the mean score and variability of ACE categories by diagnosis, and then the WERCAP score (psychosis and bipolar symptoms). The degree and direction of the linear associations between each ACE category and WERCAP score were evaluated using Pearson correlation coefficients. Using multiple linear regression, the combined influence of all ACE categories on WERCAP scores (a-WERCAP for bipolar and p-WERCAP for psychosis) was examined to determine whether ACE scores are a significant predictor of variation in these two types of symptoms. SPSS Process macro version 4.2 beta for Windows (Andrew F. Hayes; http://www.processmacro.org/download.html) was used to analyse the moderating and mediating effects of resilience on the relationship between ACEs and WERCAP. We investigated moderation by looking at how the interaction between ACEs and resilience affects WERCAP. For mediation, because we had five ACEs, we opted for a tabular form because that is more detailed.

## Results

### Frequency distribution across categories of ACEs

The most common ACE items in different categories included the following: ‘I respect myself’; ‘When I was young, I felt hated by a member or members of my family’; ‘My parents or caregivers were often drunk, stoned or wasted’; ‘When I was young, I was hit so hard that it left marks, cuts or bruises’; and ‘When I was young, someone touched me or tried to make me touch them in a sexual way’. The least common were ‘When I was young, I felt valued or important’; ‘I felt rejected by my parents or caregivers’; ‘If I needed treatment, someone would always take me to see a doctor or nurse when I was young’; ‘I think I was physically abused when I was young’; and ‘I believe that I was sexually abused when I was young’ (see Table 1).

**Table 1:** Frequency distribution across categories of adverse childhood experiences (ACEs) (*N* = 1972) Table 1 long description.

ACEs	Statement	Never	Rarely	Sometimes	Often	Nearly always	*n*
Emotional negligence	‘When I was young, I felt valued or important’^a^	680 (35.1%)	405 (20.9%)	485 (25%)	169 (8.7%)	201 (10.4%)	1940
	‘My family were emotionally warm and loving’^a^	826 (42.4%)	412 (21.2%)	411 (21.1%)	134 (6.9%)	164 (8.3%)	1947
	‘When I was young, my family looked after each other’^a^	841 (42.8%)	434 (22.1%)	321 (16.3%)	144 (7.3%)	226 (11.5%)	1966
	‘I respect myself’^a^	1227 (63.4%)	363 (18.8%)	123 (6.4%)	108 (5.6%)	113 (5.8%)	1934
	‘My family was supportive and encouraging when I was young’^a^	880 (45.3%)	343 (17.7%)	396 (20.4%)	133 (6.9%)	189 (9.7%)	1941
Emotional abuse	‘I felt rejected by my parents/caregivers’	1417 (72.4%)	160 (8.2%)	245 (12.5%)	64 (3.3%)	72 (3.7%)	1958
	‘When I was young, I was humiliated by people in my family’	1268 (64.9%)	205 (10.5%)	288 (14.7%)	102 (5.2%)	90 (4.6%)	1953
	‘I believe that I am a bad person’	1329 (67.6%)	158 (8%)	405 (20.6%)	45 (2.3%)	29 (1.5%)	1966
	‘When I was young, I felt hated by a member or members of my family’	1245 (63.8%)	212 (10.9%)	325 (16.7%)	84 (4.3%)	84 (4.3%)	1950
	‘I feel that I was put down, criticised and made to feel inferior when I was young’	1310 (67.2%)	197 (10.1%)	314 (16.1%)	71 (3.6%)	57 (2.9%)	1949
Physical negligence	‘When I was young, I felt safe and protected by somebody’^a^	1052 (53.6%)	388 (19.8%)	287 (14.6%)	119 (6.1%)	117 (6%)	1963
	‘When I was young, I was often hungry’	747 (38.2%)	415 (21.2%)	579 (29.6%)	134 (6.8%)	82 (4.2%)	1957
	‘I often had to wear ragged or dirty clothes to school’	1262 (64.7%)	250 (12.8%)	290 (14.9%)	92 (4.7%)	58 (3%)	1952
	‘My parents/caregivers were often drunk, stoned or wasted’	1516 (77.8%)	125 (6.4%)	177 (9.1%)	55 (2.8%)	76 (3.9%)	1949
	‘If I needed treatment someone would always take me to see a doctor or nurse when I was young’^a^	789 (40.5%)	357 (18.3%)	345 (17.7%)	154 (7.9%)	305 (15.6%)	1950
Physical abuse	‘When I was young, I was hit so hard that it left marks, cuts or bruises’	1128 (58%)	257 (13.2%)	346 (17.8%)	125 (6.4%)	90 (4.6%)	1946
	‘I have experienced serious physical assault’	1283 (66.3%)	259 (13.4%)	294 (15.2%)	46 (2.4%)	52 (2.7%)	1934
	‘Adults (like teachers, doctors or nurses) noticed cuts, bruises or marks from when I was beaten’	1443 (74.6%)	158 (8.2%)	221 (11.4%)	61 (3.2)	51 (2.6%)	1934
	‘I think I was physically abused when I was young’	1446 (75.4%)	143 (7.4%)	217 (11.2%)	52 (2.7%)	66 (3.4%)	1944
	‘I have been involved in life-threatening situations’	1262 (65.8%)	229 (11.9%)	307 (16%)	71 (3.7%)	50 (2.6%)	1919
Sexual abuse	‘When I was young, someone touched me or tried to make me touch them in a sexual way’	1467 (75.3%)	164 (8.4%)	207 (10.6%)	65 (3.3%)	46 (2.4%)	1949
	‘I was forced to keep secrets about someone sexually interfering with me when I was young’	1629 (83.5%)	93 (4.8%)	131 (6.7%)	52 (2.7%)	46 (2.4%)	1951
	‘I have experienced sexual assault’	1619 (83.2%)	117 (6%)	122 (6.3%)	37 (1.9%)	50 (2.6%)	1945
	‘Someone sexually molested me when I was young’	1656 (84.9%)	122 (6.3%)	96 (4.9%)	51 (2.6%)	25 (1.3%)	1950
	‘I believe that I was sexually used when I was young’	1670 (86%)	93 (4.8%)	99 (5.1%)	33 (1.7%)	46 (2.4%)	1941

a. Reversed items of ACEs. They are positively phrased questions, the scores of which are inverted during analysis so that higher values consistently indicate greater exposure to ACEs.

### Descriptive statistics of ACE and WERCAP scores

Table 2 shows the descriptive statistics for ACE and WERCAP scores, including measures of skewness. Emotional negligence exhibits the highest mean score, followed by physical negligence. Skewness values range from 0.657 to 2.476 across the ACE categories, indicating varying degrees of asymmetry in the distributions. The standard deviation range reflects the variability around the means of ACE categories. Moreover, the mean of psychosis scores surpasses that of bipolar scores.

**Table 2:** Descriptive statistics of adverse childhood experiences scores and Washington Early Recognition Center Affectivity and Psychosis (WERCAP) scores Table 2 long description.

Statistic	Emotional negligence	Emotional abuse	Physical negligence	Physical abuse	Sexual abuse	Psychotic WERCAP	Bipolar WERCAP
Mean	5.645	3.318	4.641	3.125	1.708	19.021	18.475
s.e.	0.105	0.080	0.078	0.080	0.073	0.672	0.291
Median	5.000	2.000	4.000	2.000	0	18.000	18.000
Mode	0	0	2.000	0	0	0	19.000
s.d.	4.519	3.491	3.408	3.458	3.165	13.963	7.722
Skewness	0.657	1.213	0.691	1.265	2.476	0.525	0.242
*N*	1854	1905	1896	1853	1879	432	705
95% CI	0.206	0.157	0.154	0.158	0.143	1.320	0.571

### Correlation between ACEs and WERCAP

As shown in Table 3, all ACEs positively correlate with psychosis (*P* < –0.001). All ACEs, except emotional negligence (*P* > 0.001), positively correlate with bipolar (*P* < 0.001).

**Table 3:** Correlations between adverse childhood experiences and Washington Early Recognition Center Affectivity and Psychosis (WERCAP) scores Table 3 long description.

Adverse childhood experiences	Emotional negligence	Emotional abuse	Physical negligence	Physical abuse	Sexual abuse	Bipolar WERCAP	Psychotic WERCAP
Emotional negligence	1						
Emotional abuse	0.345^**^	1					
Physical negligence	0.546^**^	0.413^**^	1				
Physical abuse	0.267^**^	0.525^**^	0.409^**^	1			
Sexual abuse	0.236^**^	0.341^**^	0.287^**^	0.450^**^	1		
Bipolar WERCAP	0.061	0.357^**^	0.188^**^	0.297^**^	0.205^**^	1	
Psychotic WERCAP	0.195^**^	0.448^**^	0.316^**^	0.352^**^	0.287^**^	0.678^**^	1

** Correlation significant at the 0.01 level (2-tailed).

### Associations between ACEs and WERCAP

As shown in Table 4, emotional and physical abuse both show a significant positive impact on a-WERCAP scores (beta 0.320, *P* < 0.001 and beta 0.140, *P* = 0.004, respectively), while emotional negligence had a negative impact (beta −0.09369, *P* = 0.042). Physical negligence and sexual abuse did not significantly impact bipolar condition. For psychosis, emotional abuse demonstrates a significant positive impact on p-WERCAP scores (beta 0.369935, *P* < 0.001), while other ACEs did not significantly contribute to the prediction.

**Table 4:** Multiple linear regression between adverse childhood experiences and scores for bipolar Washington Early Recognition Center Affectivity and Psychosis (a-WERCAP) and psychosis WERCAP (p-WERCAP) Table 4 long description.

Model	Adverse childhood experiences	Unstandardised coefficients (beta)	s.e.	Standardised coefficients (beta)	*t*	*P*-value
a-WERCAP	(constant)	15.98226	0.656517		24.344	**<0.001**
	Emotional negligence	−0.17758	0.087135	−0.09369	−2.03802	**0.042**
	Emotional abuse	0.743233	0.108099	0.320493	6.875475	**<0.001**
	Physical negligence	0.067531	0.119926	0.026994	0.563108	0.574
	Physical abuse	0.326779	0.112777	0.139507	2.897577	**0.004**
	Sexual abuse	0.128495	0.106335	0.054098	1.208399	0.227
p-WERCAP	(constant)	9.909063	1.268345	–	7.812592	**<0.001**
	Emotional negligence	−0.06597	0.170726	−0.02143	−0.3864	0.699
	Emotional abuse	1.274065	0.2079	0.369935	6.128264	**<0.001**
	Physical negligence	0.235484	0.242528	0.058746	0.970958	0.332
	Physical abuse	0.385158	0.226032	0.103824	1.703994	0.089
	Sexual abuse	0.362351	0.225567	0.088184	1.606401	0.109

Bold font denotes significant values.

### Resilience as a moderator between ACES and WERCAP (bipolar and psychosis)

The association between sexual abuse and psychosis is moderated by resilience. Nevertheless, for bipolar symptoms, this interaction was not significant (*P* = 0.786). Resilience, on the other hand, did not show moderating effects on psychosis and bipolar illness with other ACEs (see Table 5).

**Table 5:** Resilience as a moderator between adverse childhood experiences and Washington Early Recognition Center Affectivity and Psychosis (WERCAP) scores Table 5 long description.

Adverse childhood experiences	a-WERCAP (bipolar)						p-WERCAP (psychosis)					
	Beta	s.e.	95% CI		*t*	*P*	Beta	s.e.	95% CI		*t*	*P*
			Lower	Upper					Lower	Upper		
Emotional negligence												
Constant	24.045	3.24	17.682	30.409	7.422	**<0.001**	28.533	6.774	15.211	41.855	4.212	**<0.001**
Emotional negligence	−0.009	0.342	−0.68	0.663	−0.026	0.980	−0.293	0.668	−1.607	1.022	−0.438	0.662
Resilience	−0.072	0.047	−0.165	0.021	−1.519	0.129	−0.196	0.099	−0.391	−0.001	−1.975	**0.049**
Emotional negligence **×** resilience	0.001	0.005	−0.01	0.011	0.154	0.877	0.011	0.011	−0.01	0.033	1.064	0.288
Emotional abuse												
Constant	14.47	2.392	9.771	19.168	6.048	**<0.001**	9.548	4.849	0.013	19.084	1.969	**<0.05**
Emotional abuse	1.142	0.38	0.395	1.889	3.004	**0.003**	2.236	0.756	0.748	3.723	2.956	**0.003**
Resilience	0.026	0.036	−0.044	0.096	0.729	0.466	0.035	0.071	−0.106	0.175	0.488	0.626
Emotional abuse **×** resilience	−0.004	0.006	−0.016	0.009	−0.579	0.563	−0.008	0.012	−0.032	0.016	−0.68	0.497
Physical negligence												
Constant	22.636	3.149	16.451	28.821	7.188	**<0.001**	20.003	7.162	5.918	34.089	2.793	**<0.006**
Physical negligence	0.008	0.42	−0.818	0.834	0.019	0.985	0.373	0.912	−1.421	2.166	0.409	0.683
Resilience	−0.075	0.047	−0.167	0.017	−1.594	0.111	−0.127	0.105	−0.334	0.079	−1.213	0.226
Physical negligence **× r**esilience	0.006	0.007	−0.007	0.019	0.879	0.380	0.014	0.014	−0.014	0.043	0.994	0.321
Physical abuse												
Constant	19.952	2.376	15.285	24.619	8.397	**<0.001**	18.142	4.928	8.448	27.836	3.681	**<0.001**
Physical abuse	0.402	0.375	−0.334	1.138	1.072	0.284	1.39	0.818	−0.219	2.999	1.699	0.09
Resilience	−0.046	0.036	−0.117	0.024	−1.287	0.199	−0.07	0.074	−0.215	0.075	−0.946	0.345
Physical abuse **×** resilience	0.005	0.006	−0.007	0.017	0.836	0.403	−0.002	0.013	−0.028	0.024	−0.149	0.881
Sexual abuse												
Constant	22.695	1.91	18.944	26.447	11.882	**<0.001**	28.385	3.874	20.767	36.003	7.328	**<0.001**
Sexual abuse	0.34	0.445	−0.535	1.214	0.763	0.446	−1.131	1.008	−3.114	0.852	−1.122	0.263
Resilience	−0.062	0.029	−0.119	−0.005	−2.132	**0.033**	−0.185	0.059	−0.301	−0.07	−3.153	**0.002**
Sexual abuse **×** resilience	0.002	0.007	−0.012	0.016	0.272	0.786	0.038	0.017	0.003	0.072	2.153	**0.032**

Lower and upper represent the lower and upper bounds of the 95% confidence intervals, respectively. For resilience (17 items), Cronbach’s *α* was 0.904.

### Resilience as a mediator between ACEs and WERCAP (bipolar and psychosis)

For the majority of ACEs, resilience did not demonstrate a mediation effect (indirect effect, *P* > 0.05). However, the connection between emotional neglect and bipolar symptoms was fully mediated by resilience (direct effect: beta 0.042, *P* > 0.05; indirect effect: beta 0.094, *P* < 0.05). The association between sexual abuse and bipolar (direct effect: beta 0.458, *P* < 0.001; indirect effect: beta 0.046, *P* < 0.05), and that between sexual abuse and psychosis (direct effect: beta 0.986, *P* < 0.001; indirect effect: beta 0.159, *P* < 0.05), were partially mediated by resilience, as indicated in Table 6.

**Table 6:** Resilience as a mediator between adverse childhood experiences (ACEs) and Washington Early Recognition Center Affectivity and Psychosis (WERCAP) scores Table 6 long description.

Adverse childhood experiences	Effect	a-WERCAP (bipolar)						p-WERCAP (psychosis)					
		Coefficient (effect)	s.e.	*t*	*P*-value	95% CI (lower bound)	95% CI (upper bound)	Coefficient (effect)	s.e.	*t*	*P*-value	95% CI (lower bound	95% CI Upper Bound
Emotional negligence	Total effect	0.136	0.08	1.706	0.088	−0.021	0.294	0.57	0.16	3.561	**<0.001**	0.255	0.885
	Direct effect	0.042	0.089	0.474	0.636	−0.133	0.217	0.391	0.185	2.112	**0.035**	0.027	0.755
	Indirect effect	0.094	0.042		**<0.05**	0.014	0.177	0.179	0.095		>0.05	−0.017	0.359
Emotional abuse	Total effect	0.914	0.089	10.228	**<0.001**	0.738	1.089	1.737	0.169	10.269	**<0.001**	1.405	2.07
	Direct effect	0.929	0.096	9.686	**<0.001**	0.741	1.118	1.736	0.184	9.461	**<0.001**	1.376	2.097
	Indirect effect	−0.016	0.035		>0.05	−0.085	0.054	0.001	0.072		>0.05	−0.144	0.142
Physical negligence	Total effect	0.427	0.101	4.21	**<0.001**	0.228	0.626	1.322	0.202	6.537	**<0.001**	0.924	1.72
	Direct effect	0.365	0.109	3.35	**0.001**	0.151	0.579	1.25	0.227	5.52	**<0.001**	0.805	1.696
	Indirect effect	0.062	0.043		>0.05	−0.02	0.149	0.072	0.108		>0.05	−0.137	0.287
Physical abuse	Total effect	0.732	0.096	7.603	**<0.001**	0.543	0.921	1.368	0.194	7.053	**<0.001**	0.986	1.749
	Direct effect	0.703	0.101	6.99	**<0.001**	0.506	0.901	1.272	0.204	6.243	**<0.001**	0.871	1.672
	Indirect effect	0.029	0.031		>0.05	−0.032	0.094	0.096	0.064		>0.05	−0.019	0.239
Sexual abuse	Total effect	0.504	0.098	5.139	**<0.001**	0.311	0.696	1.145	0.216	5.295	**<0.001**	0.72	1.57
	Direct effect	0.458	0.1	4.596	**<0.001**	0.262	0.653	0.986	0.225	4.38	**<0.001**	0.543	1.428
	Indirect effect	0.046	0.025		**<0.05**	0.003	0.103	0.159	0.076		**<0.05**	0.027	0.325

Total effect: the total effect of ACEs on post-traumatic stress disorder (PTSD) represents the combined direct and indirect effects of ACE on PTSD. Direct effect: the direct effect of ACEs on PTSD is the effect of ACE on PTSD when the mediator is not considered. Indirect effect: the indirect effect of ACE on PTSD through resilience represents the mediated effect of ACE on PTSD through the mediator resilience.

## Discussion

### Preamble

We present the first paper from Kenya to explore the association between ACEs and WERCAP (p-WECARP and a-WERCAP), among youth living in communities in the Nairobi metropolitan area. We also highlight the role of resilience as either a mediator or moderator in the association between ACEs and WERCAP. We looked at specific categories of ACEs as described in the TADS tool, and used various statistical analyses, as described above, to derive conclusions. Our findings propose potential interventions and policy formulations for youth in the Nairobi metropolitan area.

### Frequency distribution across categories of ACEs

Our findings illustrate that emotional neglect was prevalent, with 10.4% of respondents nearly always feeling undervalued and 8.3% nearly always lacking emotional warmth and loving relationships. These results align with those of a previous study, which found that emotional neglect leads to long-term psychological issues.^10^ Emotional abuse was also evident, because 2.3% of respondents often viewed themselves as bad people, 3.3% often experienced rejection and 5.2% often felt humiliated, reflecting broader evidence that emotional abuse has lasting mental health consequences, contributing to higher risks of mood disorders and impaired self-worth, as previously documented.^24^ Our findings, that 53.6% never felt safe with somebody while 38.2% never experienced hunger, indicate a split in basic needs fulfilment among youth. In our study physical abuse was less reported, with 58% never having recorded marks or bruises and 2.4% often having faced serious assaults. This contrasts with findings from a previous study that reported higher instances of physical abuse.^25^ This variability could be due to either differences in sample characteristics or underreporting due to stigma. Regarding sexual abuse, 75.3% of respondents reported never experiencing unwanted touching, yet 2.7% were often forced into secrecy. These findings mirror those of another study which noted that, while many do not report sexual abuse, those who do often choose to remain silent.^26^ The prevalence of forced secrecy (2.7%) and other forms of sexual abuse suggests substantial underreporting among both genders, a common issue highlighted in various studies.^27^

### Descriptive statistics of ACE and WERCAP scores

Our findings show that emotional neglect had the highest mean score, making it the most prevalent ACE, followed by physical neglect, consistent with previous studies highlighting their commonality and long-term impacts.^28^ The skewness values (0.657–2.476) indicate varied asymmetry in ACE distribution, with sexual abuse showing the highest skewness (2.476), suggesting that this affects a smaller subgroup but has profound consequences.^29^ Standard deviations range from 3.165 (sexual abuse) to 4.519 (emotional negligence), reflecting considerable variability within ACE categories.

The 95% confidence intervals (0.143–0.206) indicate high precision in our mean estimates, adding reliability to the analysis. ACEs also impacted WERCAP scores, with a higher mean psychosis score (19.021) compared with bipolar (18.475), indicating a stronger link between ACEs and psychosis, consistent with previous findings on childhood adversity and severe mental health outcomes.^30^ The higher standard deviation for psychosis (13.963 *v.* 7.722 for bipolar) suggests greater variability in psychosis symptoms linked to ACEs.^31^

### Correlation between ACEs and WERCAP (bipolar and psychotic)

Our findings reveal significant associations between ACEs and both bipolar (a-WERCAP) and psychotic (p-WERCAP) symptoms. All ACEs showed a strong positive correlation with p-WERCAP (psychosis) at a highly significant level (*P* < 0.001), reinforcing the link between childhood adversity and psychotic symptoms, consistent with previous research.^32^

By contrast, all ACEs, except emotional neglect, were positively correlated with a-WERCAP (bipolar) at the same significance level (*P* < 0.001). Emotional neglect had a very weak correlation with bipolar symptoms (*r* = 0.061), differing from other ACEs and contrasting with previous studies that found a strong correlation between emotional neglect and bipolar symptoms.^33^ This highlights the need to consider the specific effects of different ACEs on mental health.

### Multiple linear regression between ACEs and a-WERCAP (bipolar) and p-WERCAP (psychotic)

Our findings show a significant positive relationship between emotional abuse and bipolar symptoms (beta 0.305, *P* < 0.001), consistent with previous studies linking emotional abuse and neglect with a worsened clinical course of bipolar symptoms.^34^ Physical abuse also shows a significant association (*P* = 0.004), aligning with previous research.^35^ Interestingly, emotional neglect demonstrates a marginally significant negative relationship with bipolar symptoms (beta −0.128, *P* = 0.042), suggesting that it may affect bipolar differently than other forms of abuse. For p-WERCAP (psychotic), emotional abuse again displays a significant positive relationship (beta 0.281, *P* < 0.001), while sexual abuse shows a marginally significant association (beta 0.092, *P* = 0.109). However, emotional neglect shows no significant relationship with psychotic symptoms (beta −0.034, *P* = 0.699), contrary to previous findings that linked emotional abuse and neglect to increased psychotic symptoms.^36^

These results suggest that emotional abuse is a key contributor to both bipolar and psychotic symptoms, while the role of sexual abuse may require further investigation because it is potentially influenced by cultural factors. The non-significant relationships with certain ACEs highlight the complexity of their impact on mental health, indicating a need for further study using mixed methods.

### Resilience as a moderator between ACES and WERCAP (bipolar and psychosis)

Our findings indicate that resilience significantly moderates the impact of sexual abuse on psychosis (*P* = 0.032), but not on bipolar, which contrasts with prior research which found that resilience moderated both.^37^ This can be explained by differences in the sociocultural interpretation and disclosure of sexual trauma in Kenyan youth, where stigma and gender norms influence both trauma response and resilience expression.^38^ Additionally, sexual abuse may activate trauma pathways more closely aligned with psychotic-like experiences than affective symptoms, potentially explaining the differential moderation.^39^ Additionally, emotional abuse, emotional neglect, physical neglect and physical abuse showed no significant moderation by resilience for either psychosis or bipolar (*P* > 0.05), consistent with other studies reporting no moderating effects of resilience on these ACEs.^40^

Notably, emotional abuse had a significant direct effect on both bipolar and psychosis (*P* = 0.003 for both), but resilience did not moderate these effects (*P* > 0.05), differing from previous studies suggesting resilience as a significant moderator.^41^

### Resilience as a mediator between ACEs and WERCAP (bipolar and psychosis)

Our findings show that resilience did not significantly mediate the effects of most ACEs (*P* > 0.05). However, resilience fully mediated the relationship between emotional neglect and bipolar (beta 0.094, *P* < 0.05), consistent with previous research.^37^ This could be due to the role of resilience in enhancing emotion regulation and social connectedness, thereby counteracting the emotional deprivation associated with neglect.^42^ Youth with higher resilience may leverage social and coping resources that buffer affective dysregulation, explaining the full mediation effect observed.^43^ Resilience also partially mediated the association between sexual abuse and WERCAP, with significant indirect effects for both bipolar and psychosis symptoms, aligning with previous studies suggesting that resilience can reduce the adverse effects of sexual abuse on mental health.^13^ In contrast, resilience did not significantly mediate the effects of emotional abuse, physical neglect or physical abuse (*P* > 0.05), a finding in accord with other research.^44^

Overall, our findings emphasise the selective protective role of resilience, particularly in the context of emotional neglect and sexual abuse. The variability found in the effects of resilience highlights the need for context-specific studies to better understand its role in mitigating the impact of different types of childhood adversity.

### Limitations

This was a cross-sectional study. A mixed-methods approach would have provided explanations for the associations and, more importantly, insights into why our findings are at variance with some of the reported studies.

Overall, our study shows a significant association between ACEs and WERCAP, including bipolar and psychotic symptoms, among youth in the Nairobi metropolitan area. Moreover, the study has highlighted the role of resilience as a moderator or mediator. Our findings are also in accord with those from global studies, emphasising that there is a general impact of ACEs on mental health. However, this study provides evidence only for Kenya, and our findings underscore the urgent need for targeted interventions addressing childhood adversity in that country. This study has filled a knowledge gap in regard to Kenya, and has also achieved both our general and specific aims.

## Data Availability

Requests for access to the data in this study should be sent to the corresponding author.

## Policy and practice recommendation

To determine who has attained a high level in developing the compilation of a single or multiple adverse childhood experiences (ACEs), resilience assessments should be incorporated into post-ACE screening and psychosocial support programmes. While resilience cannot prevent ACEs, regular assessment can help track recovery and guide interventions to strengthen adaptive coping.

## Table 1 Long description

A table with 11 rows and 7 columns detailing the frequency distribution of adverse childhood experiences (ACEs) across different categories. The columns are labeled Statement, Never, Rarely, Sometimes, Often, Nearly always, and n. The rows are grouped under Emotional neglect, Emotional abuse, Physical neglect, Physical abuse, and Sexual abuse. Each row lists a statement related to the respective category, followed by the frequency distribution percentages and the total number of responses (n). Notable trends include high percentages of 'Never' responses for statements like 'I felt rejected by my parents/caregivers' and 'I believe that I was sexually abused when I was young', and high percentages of 'Nearly always' responses for statements like 'I respect myself' and 'When I was young, someone touched me or tried to make me touch them in a sexual way'.

Navigate back to Table 1.

## Table 2 Long description

The table presents descriptive statistics for adverse childhood experiences (ACE) and Washington Early Recognition Center Affectivity and Psychosis (WERCAP) scores. It includes measures such as mean, standard error, median, mode, standard deviation, skewness, sample size, and 95 percent confidence interval. The table has 8 columns and 9 rows. Column headers are Statistic, Emotional negligence, Emotional abuse, Physical negligence, Physical abuse, Sexual abuse, Psychotic WERCAP, and Bipolar WERCAP. Row labels are Mean, s.e., Median, Mode, s.d., Skewness, N, and 95 CI. Row 1: Statistic, Emotional negligence, 5.645; Emotional abuse, 3.318; Physical negligence, 4.641; Physical abuse, 3.125; Sexual abuse, 1.708; Psychotic WERCAP, 19.021; Bipolar WERCAP, 18.475. Row 2: Statistic, Emotional negligence, 0.105; Emotional abuse, 0.080; Physical negligence, 0.078; Physical abuse, 0.080; Sexual abuse, 0.073; Psychotic WERCAP, 0.672; Bipolar WERCAP, 0.291. Row 3: Statistic, Emotional negligence, 5.000; Emotional abuse, 2.000; Physical negligence, 4.000; Physical abuse, 2.000; Sexual abuse, 0; Psychotic WERCAP, 18.000; Bipolar WERCAP, 18.000. Row 4: Statistic, Emotional negligence, 0; Emotional abuse, 0; Physical negligence, 0; Physical abuse, 0; Sexual abuse, 0; Psychotic WERCAP, 0; Bipolar WERCAP, 0. Row 5: Statistic, Emotional negligence, 4.519; Emotional abuse, 3.491; Physical negligence, 3.408; Physical abuse, 3.458; Sexual abuse, 3.165; Psychotic WERCAP, 13.963; Bipolar WERCAP, 7.722. Row 6: Statistic, Emotional negligence, 0.657; Emotional abuse, 1.213; Physical negligence, 0.691; Physical abuse, 1.265; Sexual abuse, 2.476; Psychotic WERCAP, 0.525; Bipolar WERCAP, 0.242. Row 7: Statistic, Emotional negligence, 1854; Emotional abuse, 1905; Physical negligence, 1896; Physical abuse, 1853; Sexual abuse, 1879; Psychotic WERCAP, 432; Bipolar WERCAP, 705. Row 8: Statistic, Emotional negligence, 0.206; Emotional abuse, 0.157; Physical negligence, 0.154; Physical abuse, 0.158; Sexual abuse, 0.143; Psychotic WERCAP, 1.320; Bipolar WERCAP, 0.571.

Navigate back to Table 2.

## Table 3 Long description

A table with 8 rows and 8 columns comparing correlations between adverse childhood experiences and Washington Early Recognition Center Affectivity and Psychosis (WERCAP) scores. The columns are labeled Emotional negligence, Emotional abuse, Physical negligence, Physical abuse, Sexual abuse, Bipolar WERCAP, and Psychotic WERCAP. The rows are labeled with the same categories. Each cell contains a correlation value. Row 1: Emotional negligence, 1, 0.345, 0.546, 0.267, 0.236, 0.061, 0.195. Row 2: Emotional abuse, 0.345, 1, 0.413, 0.525, 0.341, 0.357, 0.448. Row 3: Physical negligence, 0.546, 0.413, 1, 0.409, 0.287, 0.188, 0.316. Row 4: Physical abuse, 0.267, 0.525, 0.409, 1, 0.450, 0.297, 0.352. Row 5: Sexual abuse, 0.236, 0.341, 0.287, 0.450, 1, 0.205, 0.287. Row 6: Bipolar WERCAP, 0.061, 0.357, 0.188, 0.297, 0.205, 1, 0.678. Row 7: Psychotic WERCAP, 0.195, 0.448, 0.316, 0.352, 0.287, 0.678, 1.

Navigate back to Table 3.

## Table 4 Long description

A table comparing adverse childhood experiences and their impact on bipolar and psychosis scores. The table has 2 models: a-WERCAP and p-WERCAP, each with 6 rows and 6 columns. Column headers are Model, Adverse childhood experiences, Unstandardised coefficients (beta), s.e., Standardised coefficients (beta), t, and P-value. Row labels include constant, Emotional negligence, Emotional abuse, Physical negligence, Physical abuse, and Sexual abuse. For a-WERCAP, emotional abuse has a significant positive impact with a beta of 0.320493 and P-value of <0.001. Physical abuse also shows a significant positive impact with a beta of 0.139507 and P-value of 0.004. Emotional negligence has a negative impact with a beta of -0.09369 and P-value of 0.042. Physical negligence and sexual abuse do not show significant impacts. For p-WERCAP, emotional abuse has a significant positive impact with a beta of 0.369935 and P-value of <0.001. Other adverse childhood experiences do not significantly contribute to the prediction of psychosis scores.

Navigate back to Table 4.

## Table 5 Long description

The table presents data on the association between adverse childhood experiences (ACEs) and Washington Early Recognition Center Affectivity and Psychosis (WERCAP) scores, moderated by resilience. It includes two main sections: o-WERCAP (bipolar) and p-WERCAP (psychosis). Each section has columns for Beta, standard error (s.e.), 95% confidence interval (CI) with lower and upper bounds, t-value, and P-value. The rows are categorized by different types of ACEs: emotional neglect, emotional abuse, physical neglect, physical abuse, and sexual abuse. Each category includes the constant, the ACE, resilience, and the interaction between the ACE and resilience. Notable trends include significant P-values for emotional abuse and sexual abuse in the psychosis section, indicating a moderating effect of resilience. The table provides detailed statistical data to understand the relationships between ACEs, resilience, and mental health outcomes.

Navigate back to Table 5.

## Table 6 Long description

A table comparing adverse childhood experiences and their effects on Washington Early Recognition Center Affectivity and Psychosis (WERCAP) scores. The table has 12 rows and 14 columns. Column headers include Adverse childhood experiences, Effect, Coefficient (effect), s.e., t, P-value, 95% CI (lower bound), 95% CI (upper bound) for o-WERCAP (bipolar), Coefficient (effect), s.e., t, P-value, 95% CI (lower bound), 95% CI (upper bound) for p-WERCAP (psychosis). Row labels include Emotional neglect, Emotional abuse, Physical neglect, Physical abuse, Sexual abuse, each with Total effect, Direct effect, and Indirect effect. Row 1: Emotional neglect, Total effect, Coefficient (effect) 0.136, s.e. 0.08, t 1.706, P-value 0.088, 95% CI (lower bound) -0.021, 95% CI (upper bound) 0.294, Coefficient (effect) 0.57, s.e. 0.16, t 3.561, P-value <0.001, 95% CI (lower bound) 0.255, 95% CI (upper bound) 0.885. Row 2: Emotional neglect, Direct effect, Coefficient (effect) 0.042, s.e. 0.089, t 0.474, P-value 0.636, 95% CI (lower bound) -0.133, 95% CI (upper bound) 0.217, Coefficient (effect) 0.391, s.e. 0.185, t 2.112, P-value 0.035, 95% CI (lower bound) 0.027, 95% CI (upper bound) 0.755. Row 3: Emotional neglect, Indirect effect, Coefficient (effect) 0.094, s.e. 0.042, t -0.05, P-value 0.014, 95% CI (lower bound) 0.014, 95% CI (upper bound) 0.177, Coefficient (effect) 0.179, s.e. 0.095, t 1.879, P-value >0.05, 95% CI (lower bound) -0.017, 95% CI (upper bound) 0.359.

Navigate back to Table 6.
